# Identification of an Autophagy-Related Gene Signature for the Prediction of Prognosis in Early-Stage Colorectal Cancer

**DOI:** 10.3389/fgene.2021.755789

**Published:** 2021-11-25

**Authors:** Xu-tao Lin, Qiu-ning Wu, Si Qin, De-jun Fan, Min-yi Lv, Xi Chen, Jia-wei Cai, Jing-rong Weng, Yi-feng Zou, Yu-ming Rong, Feng Gao

**Affiliations:** ^1^ Department of Gastrointestinal Endoscopy, The Sixth Affiliated Hospital of Sun Yat-sen University, Guangzhou, China; ^2^ Guangdong Provincial Key Laboratory of Colorectal and Pelvic Floor Diseases, Guangdong Institute of Gastroenterology, The Sixth Affiliated Hospital of Sun Yat-sen University, Guangzhou, China; ^3^ Department of Medical Ultrasonics, The Sixth Affiliated Hospital of Sun Yat-sen University, Guangzhou, China; ^4^ Department of Colorectal Surgery, The Sixth Affiliated Hospital of Sun Yat-sen University, Guangzhou, China; ^5^ Department of VIP Region, Cancer Center of Sun Yat-sen University, Guangzhou, China

**Keywords:** colorectal cancer, prognosis, early stage, disease-free survival, autophagy-related gene

## Abstract

**Purpose:** A certain number of early-stage colorectal cancer (CRC) patients suffer tumor recurrence after initial curative resection. In this context, an effective prognostic biomarker model is constantly in need. Autophagy exhibits a dual role in tumorigenesis. Our study aims to develop an autophagy-related gene (ATG) signature-based on high-throughput data analysis for disease-free survival (DFS) prognosis of patients with stage I/II CRC.

**Methods:** Gene expression profiles and clinical information of CRC patients extracted from four public datasets were distributed to discovery and training cohort (GSE39582), validation cohort (TCGA CRC, n = 624), and meta-validation cohort (GSE37892 and GSE14333, n = 420). Autophagy genes significantly associated with prognosis were identified.

**Results:** Among 655 autophagy-related genes, a 10-gene ATG signature, which was significantly associated with DFS in the training cohort (HR, 2.76[1.56–4.82]; *p* = 2.06 × 10–4), was constructed. The ATG signature, stratifying patients into high and low autophagy risk groups, was validated in the validation (HR, 2.29[1.15–4.55]; *p* = 1.5 × 10–2) and meta-validation cohorts (HR, 2.5[1.03–6.06]; *p* = 3.63 × 10–2) and proved to be prognostic in a multivariate analysis. Functional analysis revealed enrichment of several immune/inflammatory pathways in the high autophagy risk group, where increased infiltration of T regulatory cells (Tregs) and decreased infiltration of M1 macrophages were observed.

**Conclusion:** Our study established a prognostic ATG signature that effectively predicted DFS for early-stage CRC patients. Meanwhile, the study also revealed the possible relationship among autophagy process, immune/inflammatory response, and tumorigenesis.

## Introduction

CRC is currently the second leading cause of cancer deaths worldwide, ranking third in morbidity (Bray et al., 2018). Depending upon the tumor stage at diagnosis, relative 5-year survival rates for patients with CRC range from 65% for all stages to 91, 82, and 12% for patients diagnosed with stage I, II, and IV, respectively (Miller et al., 2019). Although increasing awareness of cancer screening and advances in technology have improved early detection (Dekker et al., 2019) and enabled treatments without chemotherapy, around one-third of patients with stages I-III CRC still encounter tumor relapse after so-called curative treatment (Van Der Stok et al., 2017). Therefore, it is particularly important to identify these high-risk patients with poor prognosis. The National Comprehensive Cancer Network (NCCN) suggested such clinicopathologic features as high-risk factors for stage II colorectal cancer, including tumor size, number of lymph nodes investigated, degree of differentiation, tumor perforation, bowel obstruction, and lymphovascular invasion. However, some studies have shown that these pathological features are inadequate to accurately identify such high-risk patients (O'Connor et al., 2011; Kannarkatt et al., 2017). Accordingly, there is a growing need for novel molecular markers for prognosis patterns, which might provide valuable information for supplementary adjuvant chemotherapy or other targeted therapy. Recently, the predictive potency of KRAS and BRAF mutations, microsatellite instability (MSI) status, and CpG island methylator phenotype (CIMP) status in CRC had been studied extensively. Kim et al. (Kim et al., 2019) investigated a novel prognostic predictor based on an 11-gene signature for identifying high-risk CRC and predicting patients who will have the worst response to adjuvant chemotherapy. However, more markers await to be discovered.

Autophagy plays a dual role in tumorigenesis (Rosich et al., 2013). It inhibits early tumor initiation by the clearance of damaged mitochondria, peroxisome, and other cytotoxic substance and also caters to the high metabolic demands from accelerated proliferating tumors by degrading intracellular organelle and macromolecule substances (Kongara and Karantza, 2012; Carroll and Martin, 2013). The autophagy process includes initiation of autophagy, biogenesis of the phagophore, expansion of the phagophore, formation of the autophagosome, fusion with the lysosome, and reformation of the lysosome (Zhi et al., 2018). A few gene mutations on the autophagy process reveal correlated with human cancer. PARK2 (Parkin), an autophagy-related gene participating in mitophagy and autophagy-independent functions that regulate the cell cycle, was identified as a potential tumor suppressor on chromosome 6q25-q26 which is frequently deleted in human cancers. Autophagy substrate p62 deficiency triggered by autophagy deficiency was found to suppress tumorigenesis in mouse liver. P62 regulates NRF2 and also mTOR and NFκB, all of which are important in cancer signaling (White et al., 2015). Recent studies have started working on autophagy-related gene model building in CRC (Huang et al., 2019; Zhou et al., 2019; Cheng et al., 2021; Zhao et al., 2021). Most of them constructed different autophagy gene signatures to monitor the CRC prognosis, regardless of tumor stages. However, early-stage CRC patients may need more accurate prognostic guidance, wondering about the need for additional chemotherapy. So, to further investigate how autophagy affects the prognosis of early-stage CRC patients, we identified an autophagy-related gene (ATG) signature from CRC-specific transcriptomes based on high-throughput data analysis. The ATG signature, which stratified the stage I/II CRC patients into distinct risk groups, together with functional analysis, might provide insights into the mechanism of CRC recurrence and targeted treatment.

## Methods

### Public Datasets

Gene expression data of CRC tissue samples and corresponding clinical information obtained from the public database were retrospectively analyzed. A total of 1,610 patient samples from four independent public cohorts were included. Three hundred nine patients without adjuvant chemotherapy but with survival information in the GSE39582 dataset (n = 566) served as the discovery cohort; stage I and II patients among these 309 patients were intended for training. The Cancer Genome Atlas (TCGA) CRC dataset (n = 624) was used for independent validation, while the GSE37892 and GSE14333 datasets (n = 420) were combined for meta-validation. All datasets are from Affymetrix Human Genome U133 Plus 2.0 Array. TCGA cohort data were downloaded from Broad GDAC Firehose before transcripts per million (TPM) of level 3 RNA-Seq data in the log2 scale were applied to calibrate the gene expression levels. Other datasets were obtained directly in their processed format from the Gene Expression Omnibus database (GEO) through the Bioconductor package “GEOquery.” The “combat” algorithm of the R package “sva” and the z-scores were used to correct the batch effects, so as to standardize microarray data across multiple experiments and compare them independent of the original hybridization intensities.

### Construction of a Prognostic ATG Signature

The absolute median difference (MAD) was used to select the ATGs (Guinney et al., 2015). MAD is a robust statistic of statistical divergence that is more adaptable to outliers in a dataset than the standard deviation. To construct a prognostic ATG signature, we focused on 655 ATGs from eight gene sets identified *via* MSigDB (version 6.2) (Subramanian et al., 2005; Liberzon et al., 2011; Liberzon et al., 2015) with the keyword “autophagy.” Only 617 genes measured on all platforms involved in this study with high variation (MAD >0.5) were selected. After 1,000 times random Cox univariate regressions, genes with repeated significance, which indicated a strong relationship between ATGs and patients’ disease-free survival (DFS), were selected as candidates for the signature. A Cox proportional hazard regression model on CRC samples together with the least absolute shrinkage and selection operator (LASSO) was applied to minimize the risk of overfitting as well as to generate a risk model.

Patients were divided into low and high autophagy risk groups in accordance with the optimal ATG signature cutoff, which was defined by the time-dependent receiver operating characteristic (ROC) curve analysis at 5 years of DFS in the training cohort. The ATG signature score with the largest Youden’s index in the ROC curve was deemed as the cutoff value.

### Validation of ATG Signature

Oncotype DX is a quantitative multi-gene, real-time polymerase chain reaction (RT-PCR) assay that measures gene expression in paraffin-embedded tumor tissues. The C-index was employed in the GSE39582 cohort, TCGA cohorts, and meta-validation cohorts respectively in comparison to Oncotype DX colon to assess the predictive capability of the model. For further validation, the prognostic value of the ATG signature was evaluated in CRC patients with early stages (stage I and II) and all stages in different cohorts through survival analysis. Univariate and multivariate analyses of the ATG signature and available clinical parameters were performed to assess whether the ATG signature is an independent risk factor. The independent risk factors identified by multivariate Cox regression analysis were applied to construct the nomogram for estimating the DFS of 5 years in CRC.

### Functional Analysis

Enrichment of potential pathways of the ATG signature by gene ontology (GO) analysis was performed on gProfiler (https://biit.cs.ut.ee/gprofiler/), and gene set enrichment analysis (GSEA) (Newman et al., 2015) was conducted using the Bioconductor package “fgsea.” Gene sets of cancer hallmarks from MSigDB with statistical significance (FDR-adjusted *p*<0.05) were selected (Markle et al., 2010). CIBERSORT (Mokarram et al., 2017) was used to dissect immune cell infiltration in different risk groups.

### Statistical Analysis

All statistical analyses were performed in R software (version 3.5.1). Categorical variables were reported as count. Continuous data were reported as mean with standard deviation (SD) and compared with the Student’s t-test. The LASSO regression was plotted using the “glmnet” R package (version: 2.0-16). Time-dependent ROC curve analysis was done by the R package “survivalROC” (version: 1.0.3). Survival analysis was conducted using the Kaplan–Meier method and compared with the log-rank test. Univariate and multivariate analyses of ATG signature and clinical parameters were performed using the log-rank test. The statistical significance level was set at *α* = 0.05, two-sided.

## Results

### ATG Signature Establishment

**FIGURE 1:**
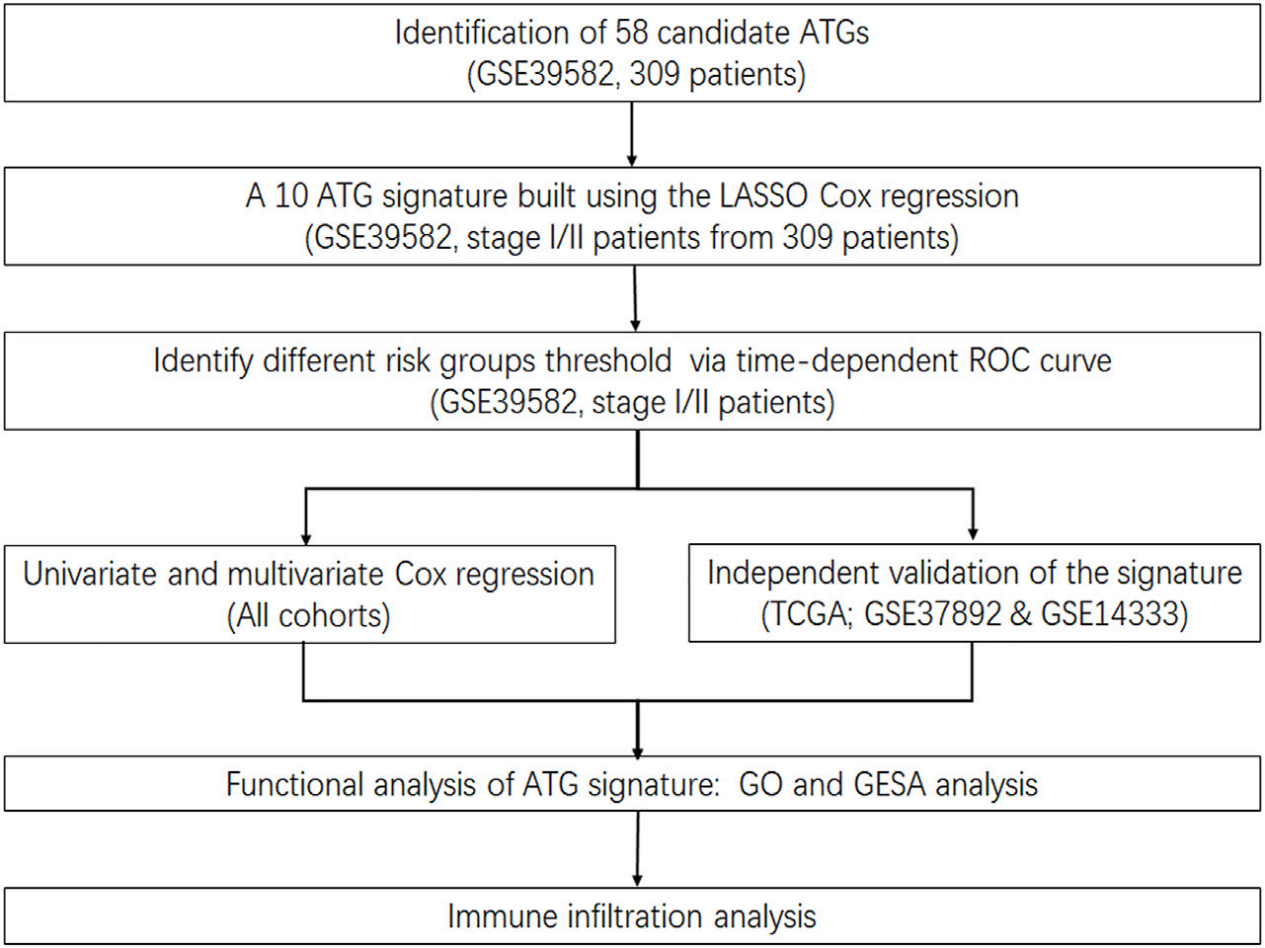
Flowchart.

**FIGURE 2:**
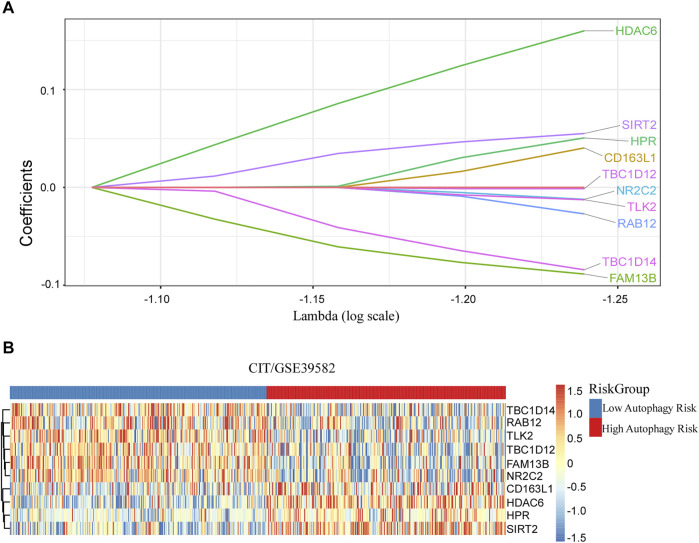
Discovered ATG signature for prognostic prediction of colorectal cancer. **(A)** Ten ATGs signatures were found from the LASSO Cox regression. **(B)** Heatmap of the identified 10-gene signature.

After filtration with MAD >0.5, 617 genes measured on all platforms were selected for this study. By 1,000 times random Cox univariate regressions, 58 ATGs were identified to be strongly relevant to DFS and considered as candidates for the signature (Figure 1). A LASSO Cox regression in stage I and II patients in the training cohort (Table 1) revealed 10 ATGs for the risk model (Figure 2), with the coefficient of each ATG listed in Table 2. The risk model was formulated as follows: autophagy-related risk score = 0.040346631 × exp CD163L1 + 0.040346631 × exp FAM13B + 0.160103165 × exp HDAC6 + 0.05063732× exp HPR − 0.012205947 × exp NR2C2 − 0.027104325 × exp RAB12 + 0.055095935 × exp SIRT2 − 0.084226324 × exp TBC1D14 − 0.012613054 × exp TLK2 − 0.001301898 × exp TBC1D12 (each gene represents its mRNA expression level).

**TABLE 1:** Characteristics of GSE39582, TCGA, and meta-validation cohorts.

	GSE39582 (discovery and training)	TCGA (validation)	Meta-validation
Number of patients	566	624	420
Patients with survival data	557	509	356
Mean age, years	66.85 ± 13.29	66.27 ± 12.76	66.66 ± 12.60
Gender, n			
Male	310	332	233
Female	256	292	187
TNM stage, n			
Stage I	33	105	44
Stage II	264	230	167
Stage III	205	180	148
Stage IV	60	88	61
NA	4	21	-
CMS system, n			
CMS1	91	68	74
CMS2	232	207	168
CMS3	69	64	69
CMS4	127	117	97
NA	47	168	12
Tumor location			
Left	342	354	233
Right	224	270	185
NA	—	—	2
RFS event, n			
Yes	177	100	87
No	380	416	269
NA	9	108	64
OS event, n			
Yes	191	67	NA
No	371	557	NA
NA	4	—	420
DFS event, n			
Yes	248	146	87
No	314	386	269
NA	4	92	64
MMR status, n			
MSI	75	189	-
MSS	444	431	-
NA	47	4	420
CIMP status, n			
Positive	91	—	—
Negative	405	—	—
NA	70	624	420
CIN status, n			
Positive	353	—	—
Negative	110	—	—
NA	103	624	420
TP53 status, n			
Wild type	161	—	—
Mutation	190	—	—
NA	215	624	420
KRAS status, n			
Wild type	328	34	—
Mutation	217	30	—
NA	21	560	420
BRAF status, n			
Wild type	461	32	—
Mutation	51	3	—
NA	54	589	420
Chemotherapy adjuvant, n			
Yes	233	231	118
No	316	393	171
NA	17	—	131

RFS, relapse-free survival; OS, overall survival; DFS, disease-free survival; MMR, mismatch repair; CIMP, CpG island methylator phenotype; CIN, chromosomal instability; NA, not available.

**TABLE 2:** Model information.

Gene	Name	Frequency in resampling	Average *p*-value	Coefficient
CD163L1	CD163 molecule-like 1	674	0.050594905	0.040346631
FAM13B	Family with sequence similarity 13 member B	625	0.050594905	0.040346631
HDAC6	Histone deacetylase 6	719	0.042438619	0.160103165
HPR	Haptoglobin-related protein	769	0.051952291	0.05063732
NR2C2	Nuclear receptor subfamily 2 group C member 2	898	0.019300065	-0.012205947
RAB12	RAB12, member RAS oncogene family	866	0.025407812	-0.027104325
SIRT2	Sirtuin 2	877	0.023493248	0.055095935
TBC1D14	TBC1 domain family member 14	998	0.002105817	-0.084226324
TLK2	Tousled like kinase 2	743	0.040418795	-0.012613054
TBC1D12	TBC1 domain family member 12	999	0.002072649	-0.001301898

**FIGURE 3:**
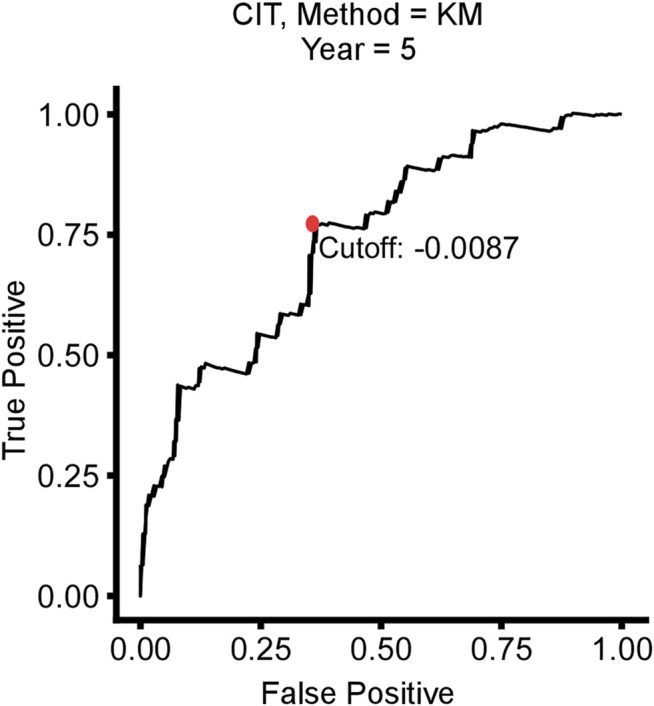
Time-dependent ROC curve for 5-year disease-free survival (DFS) in stage I/II CRC patients in the training cohort.

**FIGURE 4:**
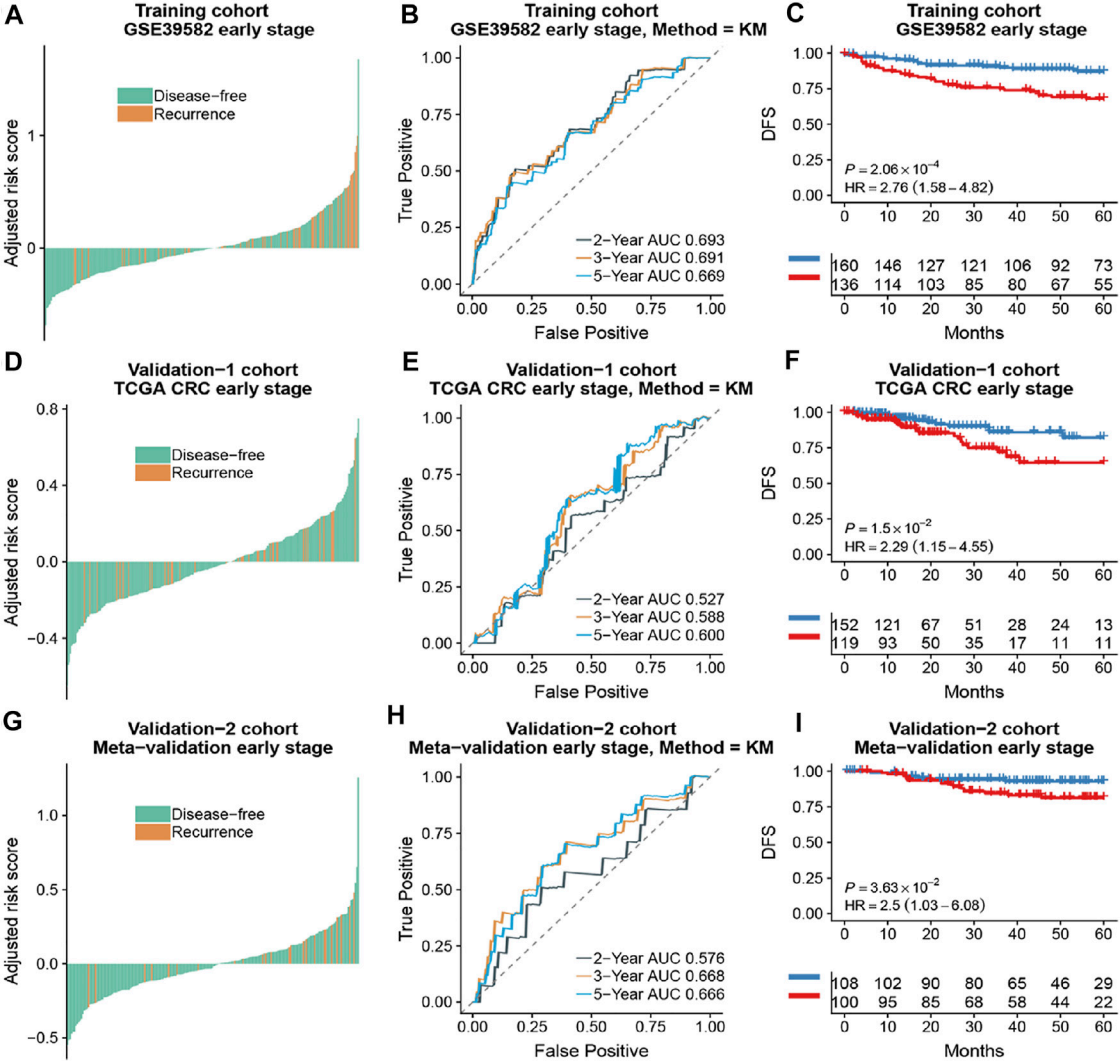
The association of the ATG signature with DFS in early-stage (stage I and II) patients with CRC. Patients with CRC of the early stage were ranked by autophagy risk scores in the training cohort **(A)**, the TCGA cohort **(D)**, and the meta-validation cohort **(G)**. Time-dependent ROC curves for DFS in early stage (stage I and II) patients achieved with different durations in the training cohort **(B)**, the TCGA cohort **(E)**, and the meta-validation cohort **(H)**. Kaplan–Meier curves showed DFS of early-stage patients in low and high autophagy groups in the training cohort **(C)**, the TCGA cohort **(F)**, and meta-validation cohort **(I)**, respectively. *p* values comparing risk groups were calculated with the log-rank test. Hazard ratios (HRs) and 95% CIs are for low vs. high autophagy risk. CRC, colorectal cancer; ATG, autophagy-related gene; DFS, disease-free survival.

**FIGURE 5:**
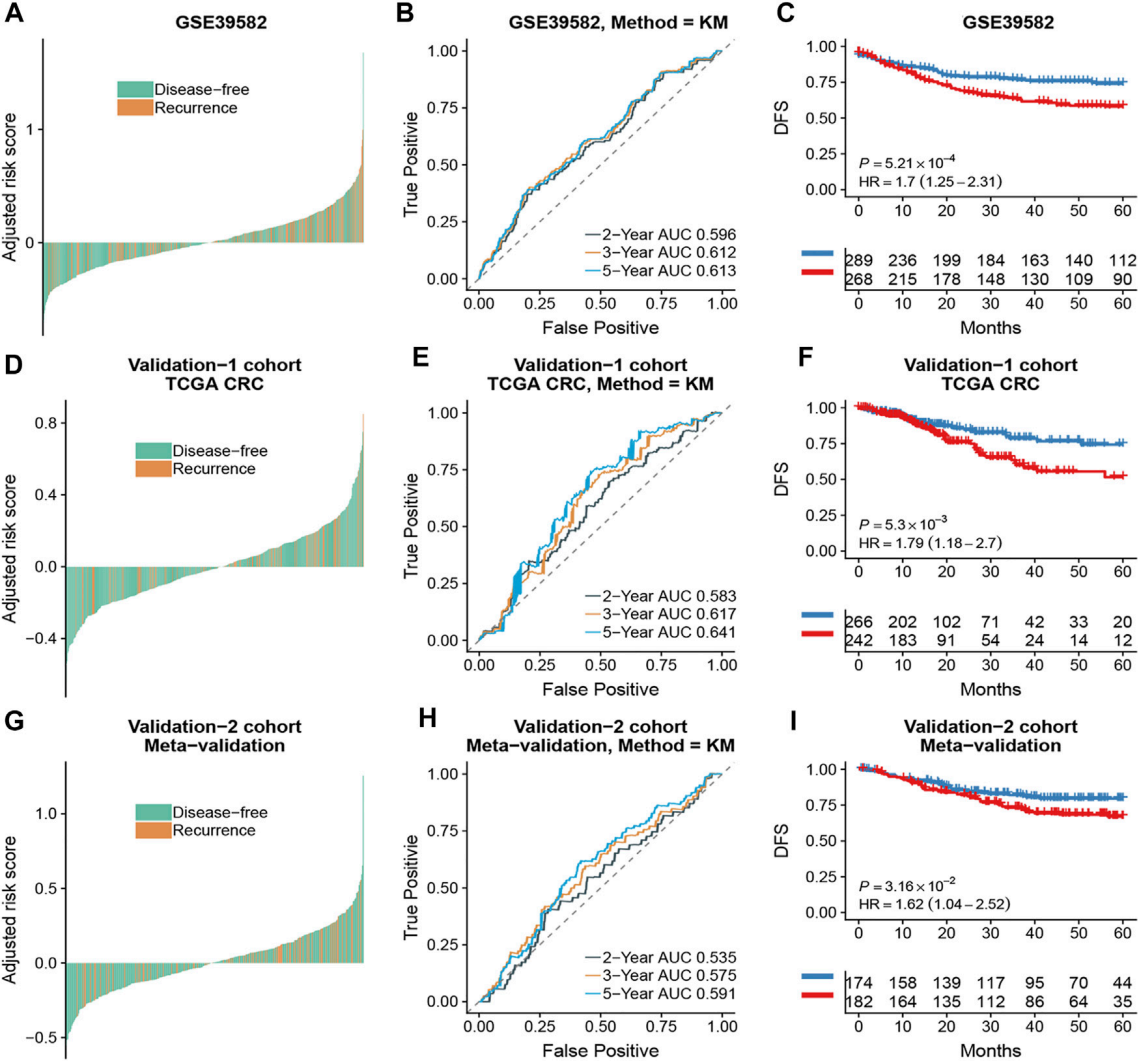
The association of the ATG signature with DFS in CRC patients with all stages. Patients with CRC of the early stage were ranked by autophagy risk scores in the GSE39582 cohort **(A)**, the TCGA cohort **(D)**, and the meta-validation cohort **(G)**. Time-dependent ROC curves for DFS in CRC patients were achieved with different durations in the GSE39582 cohort **(B)**, the TCGA cohort **(E)**, and the meta-validation cohort **(H)**. Kaplan–Meier curves showed DFS of CRC patients in low and high autophagy groups in the GSE39582 cohort **(C)**, TCGA cohort **(F)**, and meta-validation cohort **(I)**, respectively. *p* values comparing risk groups were calculated with the log-rank test. Hazard ratios (HRs) and 95% CIs are for low vs. high autophagy risk. CRC, colorectal cancer; ATG, autophagy-related gene; DFS, disease-free survival.

Based on the time-dependent ROC curve analysis of 5-year DFS in the training cohort, the optimal cutoff of ATG signature that divided the patients into high and low autophagy risk groups was −0.0087 (Figure 3). The risk scores of all patients are shown in Supplementary Table S1. Survival analysis showed that the DFS rate was higher in the low autophagy risk group compared to the high autophagy risk group for patients with early stages (stages I and II) in the training cohort (Figures 4A–C, HR, 2.76[1.56–4.82]; *p* = 2.06 × 10–4). So it was indicated for patients in all stages in the GSE39582 dataset (Figures 5A–C, HR, 1.7[1.25–2.31]; *p* = 5.21 × 10–4).

### Validation of the ATG Signature

To assess the predictive capability of the risk model, the C-index was first applied to various cohorts which turned out to be 0.74 (95% CI, 0.63–0.85) in the GSE39582 cohort, 0.70 (95% CI, 0.54–0.85) in the TCGA cohort, and 0.70 (95% CI, 0.51–0.89) in the meta-validation cohort (Table 3), higher than those of Oncotype DX colon. We employed the same formula to the independent validation cohort (TCGA) and the meta-validation cohort (GSE37892 and GSE14333). Patients were significantly stratified into different risk groups by the ATG signature considering DFS. For early stages, CRC patients in the high autophagy risk group displayed poorer DFS in both the independent validation cohort (Figures 4D–F, HR, 2.29[1.15–4.55]; *p* = 1.5 × 10–2) and the meta-validation cohort (Figures 4G–I, HR, 2.5[1.03–6.06]; *p* = 3.63 × 10–2). So it was indicated for patients with all stages in both the independent validation (Figures 5D–F, HR, 1.79[1.16–2.7]; *p* = 5.3 × 10–3) and meta-validation cohorts (Figures 5G–I, HR, 1.64[1.04–2.52]; *p* = 3.16 × 10–2). Besides, the univariate and multivariate analyses further proved ATG as an independent prognostic factor after adjusting for clinical parameters such as sex and tumor stage (Table 4). Nomogram is displayed in Supplementary Figure S1.

**TABLE 3:** C-index for autophagy risk compared with Oncotype DX colon in three cohorts.

Cohorts	Autophagy risk		Oncotype DX colon	
	C-index	95% CI	C-index	95% CI
GSE39582	0.74	0.63–0.85	0.65	0.53–0.77
TCGA	0.70	0.54–0.85	0.61	0.44–0.77
Meta-validation	0.70	0.51–0.89	0.62	0.43–0.82

TCGA, The Cancer Genome Atlas.

**TABLE 4:** Univariate and multivariate analyses of ATG signature and clinical and pathologic factors in validation cohorts.

Characteristic	GSE39582				TCGA				Meta-validation			
	Univariate		Multivariate		Univariate		Multivariate		Univariate		Multivariate	
	HR (95%CI)	*p*	HR (95%CI)	*p*	HR (95%CI)	*p*	HR (95%CI)	*p*	HR (95%CI)	*p*	HR (95%CI)	*p*
ATG signature	2.76 (1.58–4.82)	0.00021	2.66 (1.52–4.65)	0.0006	2.27 (1.14–4.51)	0.016	2.27 (1.14–4.51)	0.016	2.50 (1.03–6.08)	0.036	2.50 (1.03–6.08)	0.036
Gender	1.51 (0.87–2.61)	0.14			1.29 (0.65–2.53)	0.46			0.86 (0.38–1.96)	0.73		
Age	1.01 (0.99–1.03)	0.4			1.01 (0.98–1.04)	0.46			0.97 (0.94–1.00)	0.08		
Tumor location	1.09 (0.63–1.88)	0.75			0.94 (0.48–1.83)	0.85			1.28 (0.55–2.96)	0.56		
TNM stage	7.61 (1.07–53.86)	0.016	7.19 (1.01–51.32)	0.049	1.74 (0.72–4.20)	0.22			2.67 (0.63–11.38)	0.17		
MMR status	1.60 (0.68–3.75)	0.28			0.78 (0.40–1.54)	0.47						
CIMP positive	0.99 (0.46–2.11)	0.97										
CIN positive	1.60 (0.71–3.62)	0.26										
TP53 mutation	1.40 (0.77–2.54)	0.26										
KRAS mutation	1.26 (0.74–2.13)	0.4			0.82 (0.17–4.07)	0.81						

ATG, autophagy-related gene; MMR, mismatch repair; CIMP, CpG island methylator phenotype; CIN, chromosomal instability; HR, hazard ratio; CI, confident interval.

### Functional Analysis of the ATG Signature

**FIGURE 6:**
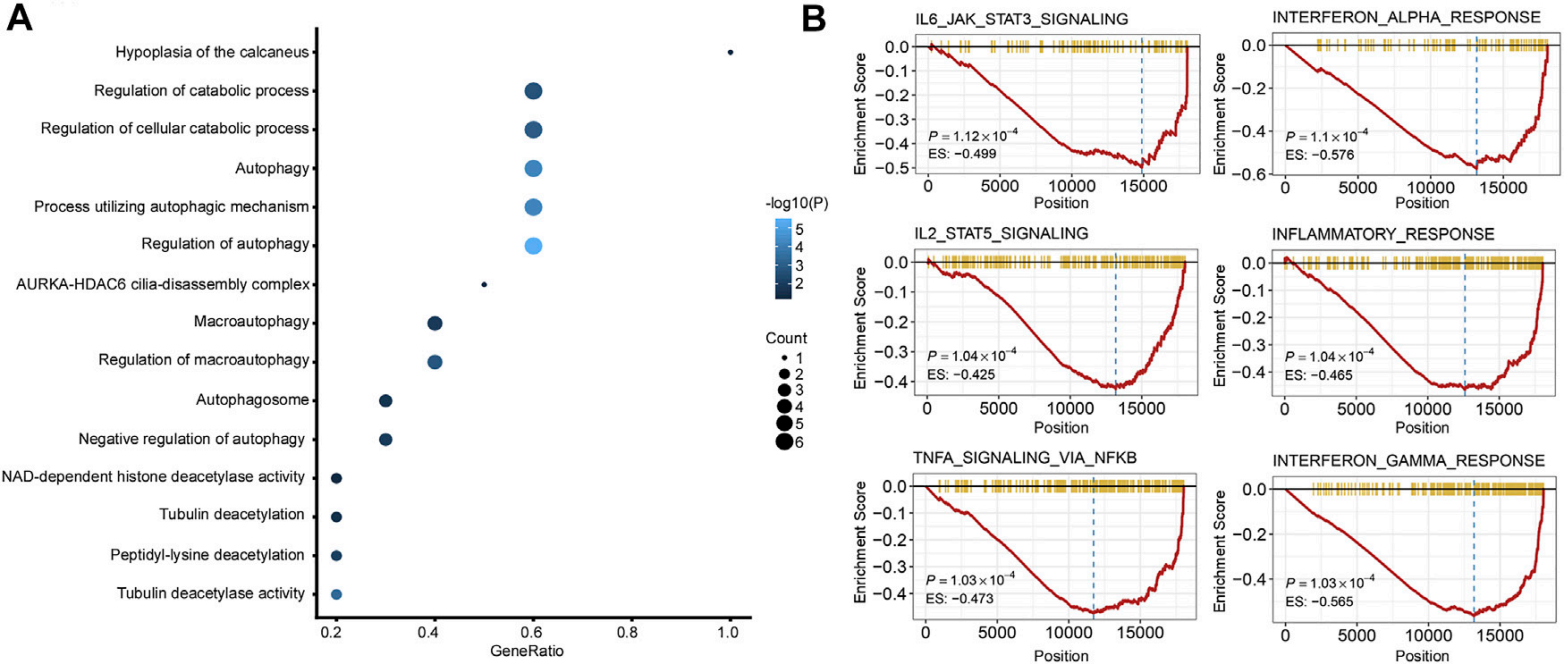
Functional analysis of the 10 autophagy signature genes. **(A)**. Gene ontology (GO) of the 10 autophagy signature genes. **(B)**. GSEA showed some immune/inflammatory pathways downregulated in high autophagy risk patients.

GO analysis and GSEA were carried out to explore the biological function and signaling pathways of genes from the ATG signature. GO analysis revealed that the genes within the ATG signature were mostly involved in the regulation of autophagy and catabolic processes (Figure 6A; Supplement Table S2). GSEA was performed between different risk groups to further investigate the pathways that were significantly affected. We found a significant enrichment in multiple immune/inflammatory pathways in the high autophagy risk group, including the IL6/JAK/STAT3 signaling pathway, the IL2/STAT5 pathway, the IFN-α pathway, the IFN-γ pathway, and the inflammatory response pathway (Figure 6B, *p* value < 0.005). Some cell cycle/metabolism-associated pathways, including G2-M, oxidative phosphorylation, E2F, and MYC, were also significantly enriched in the high autophagy risk group, in addition to a few classic pathways like mTORC1 and epithelial–mesenchymal transmission (EMT; *p* value < 0.005).

**FIGURE 7:**
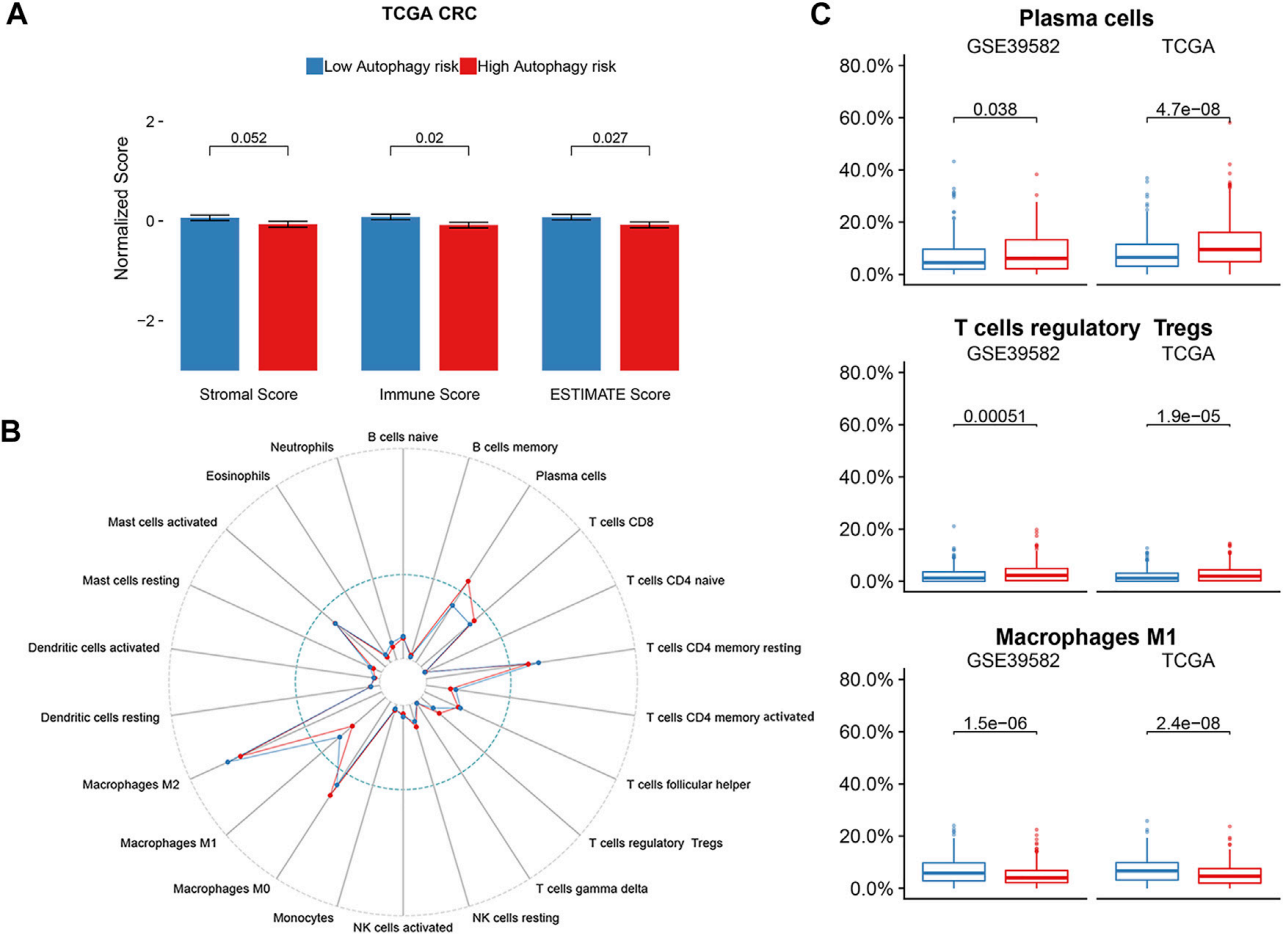
Immune infiltration status of ATG signature risk groups. **(A)** ESTIMATE algorithm in the TCGA cohort. **(B)** Twenty-two immune cells abundance for different immune risk groups. **(C)** Plasma cells and T cells regulatory were enriched in the high-risk group. The macrophage M1 was enriched in the low-risk group. *p*-values are based on the Wilcoxon test. Hazard ratios (HRs) and 95% CIs are for low vs. high autophagy risk. CRC, colorectal cancer; DFS, disease-free survival.

As there was a significant enrichment in the immune/inflammatory pathway through GSEA analysis, we conducted immune infiltration analysis. The ESTIMATE algorithm displayed significant differences in the immune score (*p* = 0.02) and ESTIMATE score (*p* = 0.027) between the high and low autophagy risk groups in the TCGA CRC cohort (Figure 7A). Infiltration of plasma cells and Tregs was enriched significantly in the high autophagy risk group compared with the low autophagy risk group in the GSE39582 and TCGA cohorts (Figure 7B–C). By contrast, M1 macrophage infiltration turned out to be significantly lower in the high autophagy risk group (Figure 7B–C). Similar results are shown in Supplementary Figure S2.

### Adjuvant Chemotherapy Effects on Different Autophagy Risk Groups

**FIGURE 8:**
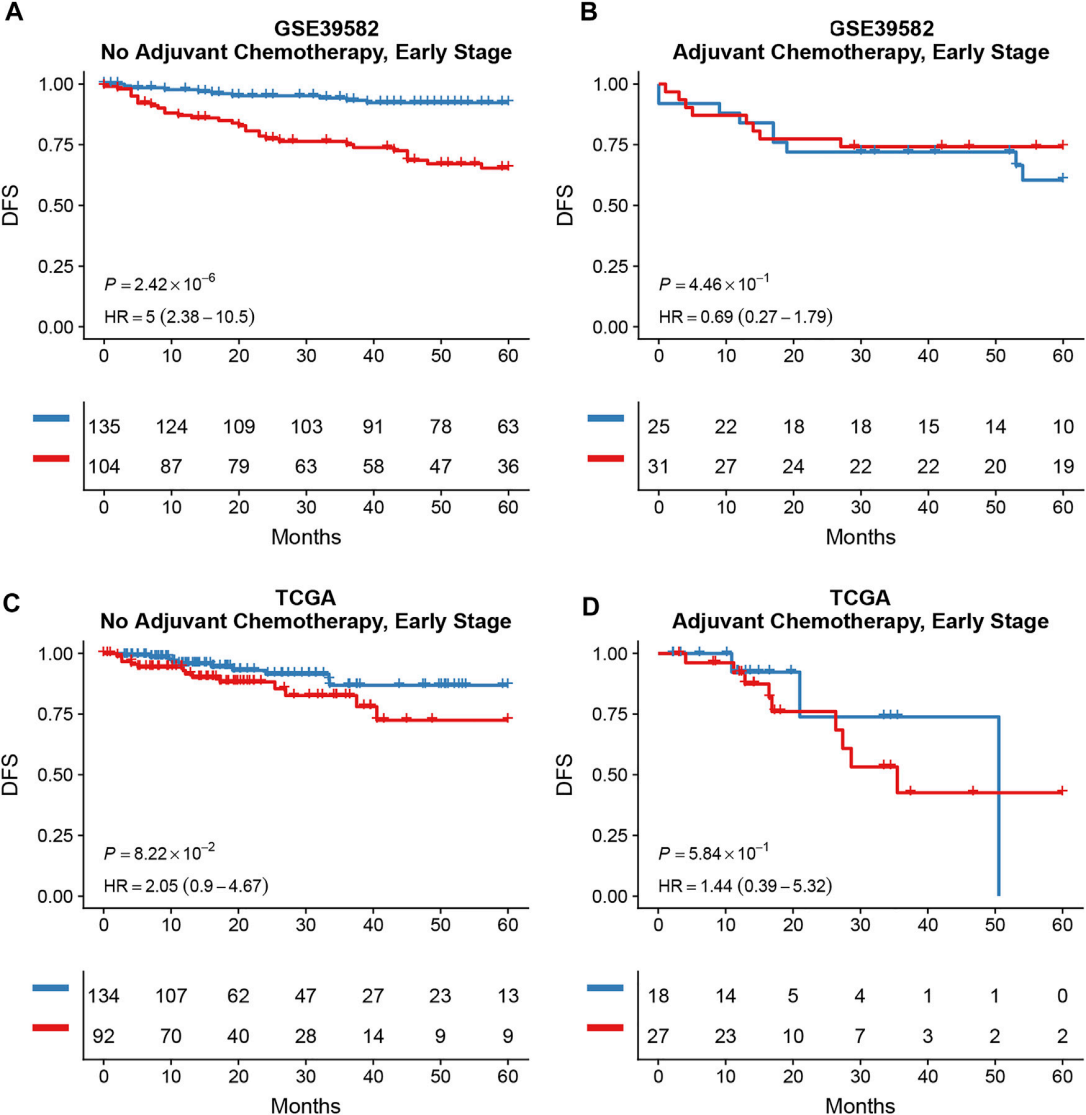
Effects of adjuvant chemotherapy on DFS between different autophagy risk groups in early stage (stage I and II) patients with CRC. Kaplan–Meier curves showed DFS of early-stage patients between low and high autophagy groups with and without adjuvant chemotherapy respectively in the GSE39582 **(A,B)** cohort and the TCGA cohort **(C,D)**. *p* values comparing risk groups were calculated with the log-rank test.

**FIGURE 9:**
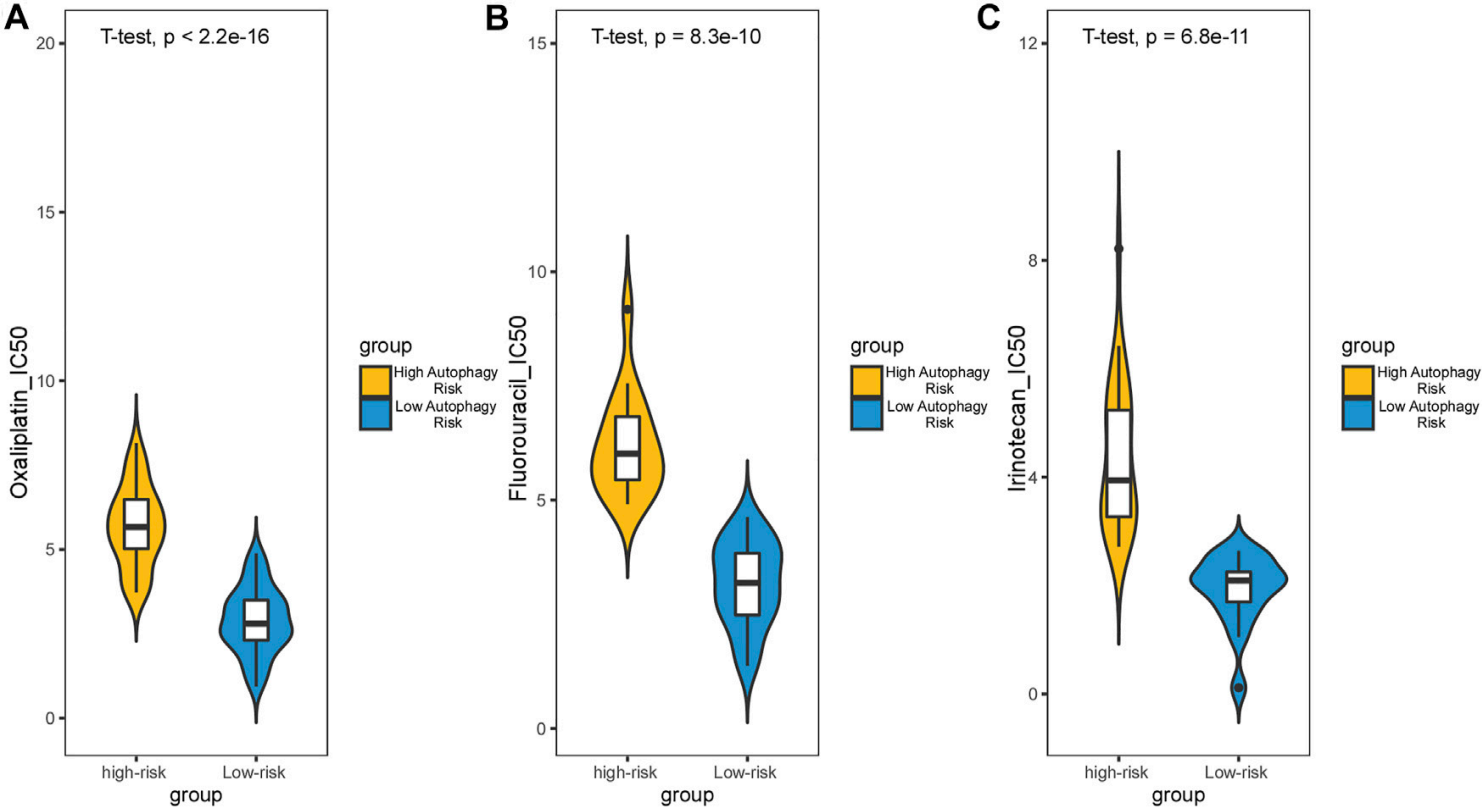
Effect of chemotherapy drugs on colorectal cancer cell lines from different autophagy risk groups. IC-50 of oxaliplatin **(A)**, fluorouracil **(B)**, and irinotecan **(C)** were lower in the low autophagy risk group. *p* values were calculated by T-test.

Survival analysis conducted for different autophagy risk groups with and without adjuvant chemotherapy respectively showed that for early-stage CRC patients without adjuvant chemotherapy in the high autophagy risk group displayed poorer DFS in the GSE39582 cohort (Figures 8A, HR, 5[2.38–10.5]; *p* = 2.42 × 10–6). However, DFS showed no significant difference between high and low autophagy risk groups for early CRC patients with adjuvant chemotherapy (Figure 8B, *p* = 4.46 × 10–1). Similar results were observed in the TCGA cohort (Figure 8C, HR, 2.05[0.9–4.67]; *p* = 8.22 × 10–2 and Figure 8D, *p* = 5.84 × 10–1). In addition, according to the cutoff values, we divided 48 kinds of cell lines related to colorectal cancer into high and low autophagy risk groups to test the effects of different chemotherapy drugs on cell lines and found that the half-maximal inhibitory concentrations (IC-50) of oxaliplatin, fluorouracil, and irinotecan were lower in the low autophagy risk group (Figures 9A–C). These results indicate that the model can be used to predict the drug sensitivity of cell lines to chemotherapeutic drugs under different autophagy risk groups.

## Discussion

Thanks to the improved awareness of cancer screening, CRC is now detected at an early stage, resulting in a better rate of survival. Surgery without chemotherapy, which was deemed as the curative treatment, was carried out on the majority of patients with stage I/II colon cancer and in some cases of stage I/II rectal cancer (Miller et al., 2019). Indeed, it enabled prevention from unnecessary side effects of chemotherapy. Nevertheless, more than 20% of patients with stage I/II CRC who underwent surgical resection still suffered recurrence (Markle et al., 2010). Quite a few multigene prognostic signatures have been developed for CRC, but none of them graduated to the widespread application due to the uncertainty of prognostic accuracy. Accordingly, an effective prognostic model composed of multiple biomarkers to distinguish early-stage patients with a high risk of recurrence is crucial and necessary for elective adjuvant chemotherapy or other targeted treatments.

Emerging studies have revealed that autophagy functions diversely in the development, maintenance, and progression of tumors. While autophagy may prevent cellular cancerous transformation in normal tissue, it acts as a survival mechanism in established tumors, especially under stress conditions and in response to chemotherapy (Mokarram et al., 2017). Several autophagy inhibitors and activators have been brought up as improved chemotherapeutic options for cancer treatment (Koustas et al., 2019), but without sufficient clinically significant results, especially in CRC. Accordingly, further investigation on the biological mechanism of autophagy in the tumor microenvironment deserves attention, and more targets associated with autophagy await to be found.

In our study, we developed a prognostic model comprised of 10 ATGs for stage I/II CRC. This ATG signature, which classified patients into high and low autophagy risk groups, demonstrated a significant difference in 5-year DFS. The C-index of the ATG signature exhibited a good clinical predicting fitness superior to the Oncotype DX colon. Validation results suggested that the ATG signature could successfully predict DFS for stage I/II CRC patients after treatment. This novel model enabled us to identify CRC patients with high autophagy risk which stood for increased risk of tumor recurrence. Surprisingly, despite the original intention for DFS prediction of early-stage CRC patients, the ATG signature also showed a significant effect in the prediction for all stages. Therefore, this model could be applied to predict tumor recurrence in all CRC patients regardless of tumor stage.

As we looked over the genes within the ATG signature, some of them have been found correlated with CRC but mostly bear context-dependent biological functions in cancers, similar to autophagy. For example, the cytosolic histone deacetylase 6 (HDAC6) served as a tumor suppressor in hepatocellular carcinogenesis (Yang et al., 2019), while another study revealed that the HDAC6 inhibitor significantly suppressed the proliferation and viability and induced apoptosis in CRC cells, where autophagy activation was observed (Chen et al., 2019). Elevated Sirtuin 2 (SIRT2) was found to be associated with poor prognosis in CRC patients *via* its participation in tumor angiogenesis (Hu et al., 2019). Meanwhile, in a separate study SIRT2 was found to be downregulated in colon cancer biopsies compared to adjacent noncancerous tissues, and overexpression of SIRT2 inhibited the proliferation and metastatic progression of SW480 cells (Zhang et al., 2017). In terms of autophagy-related functions, a previous investigation reported that in response to oxidative stress or serum starvation, SIRT2 dissociated as acetylated FOXO1, which later bound to autophagy protein 7 (ATG7) and induced autophagy in tumors (Zhao et al., 2010). As these inconsistencies make it difficult to clarify the role of autophagy in CRC, we further investigated the biological functions of the ATG signature, expecting to find some clues in the autophagy-related functions in tumors.

GSEA revealed that the ATG signature included genes that were robustly involved in multiple immune/inflammatory pathways including IL6/JAK/STAT3, IL2/STAT5, IFN-α, IFN-γ, and TNF-α/NF-κB, and the inflammatory response presented a particular relation to CRC proliferation or prognosis as previous studies revealed (Nichols et al., 1994; Eguchi et al., 2003; De Simone et al., 2015; Park et al., 2017; Giordano et al., 2019). As our findings suggested that a high autophagy risk score correlated with the downregulation of these immune/inflammatory pathways, we speculated that autophagy might play a role in CRC tumorigenesis and tumor proliferation *via* an anti-immune/anti-inflammatory response. Moreover, increased infiltration of Tregs and decreased infiltration of M1 macrophages observed in the high autophagy risk group during immune infiltration analysis seemingly catered to the anti-immune/anti-inflammatory response. Tregs are known to suppress both antibody-mediated and cell-mediated immune responses (Wing and Sakaguchi, 2010). The pro-inflammatory M1 macrophages, a phenotype of tumor-associated macrophages (TAMs), correlated with a better prognosis in CRC patients for its tumor-suppressing function (Edin et al., 2012; Shapouri-Moghaddam et al., 2018). By triggering an anti-immune or anti-inflammatory response, autophagy might promote polarization or re-polarization toward the M2 phenotype spontaneously and thus lead to the decrease of M1 infiltration observed. Previous studies have described the link between autophagy and macrophage polarization in the tumor microenvironment. For example, mTOR which functions as a conserved kinase protein in the regulation of autophagy also participates in the polarization of monocytes into TAMs (Chen et al., 2014). More new strategies targeting TAM polarization as well as autophagy await exploration, and further studies are needed to clarify the above speculations.

Upon the prognostic prediction, clinicians could thus make an informed decision regarding supplementary treatment regimens. For example, we can selectively apply more aggressive chemotherapy strategies to early CRC patients of high autophagy risk groups. As our statistics suggest, DFS showed no significant difference between high and low autophagy risk groups for early CRC patients with adjuvant chemotherapy. However, the predicted results of the IC-50 of the colorectal cancer-associated cell lines showed a higher sensitivity to chemotherapy in the low autophagy risk group. This may be due to the limitations of retrospective studies, in which clinicians choose chemotherapy based on the patient’s condition rather than random assignment. Cell experiments can reduce heterogeneity. It is hard to state whether those antitumor agents of chemotherapy are inhibiting autophagy or not. However, combined with our findings that a high autophagy risk score correlated with the downregulation of these immune/inflammatory pathways, chemotherapy may prevent tumor proliferation or recurrence by triggering specific immune/inflammatory responses or modifying the tumor microenvironment through TAMs. Besides, it is possible that chemotherapy just reduces the impact of autophagy on tumor relapse through massive, indiscriminate cell killing, and immunosuppression. Possible mechanisms will be explored in the further basic experiment and prospective clinical studies.

However, our prognostic ATG signature relies on the gene expression profiles from microarray platforms, which makes it too expensive and time-consuming to popularize in clinical application. In addition to the dataset limitations from retrospective studies, further prospective clinical tests are recommended to validate our results. Despite the limitations, our research proposes a novel perspective to predicting the prognosis of early CRC patients and offers valuable insights into the relationship between autophagy, immune/inflammatory response, and tumorigenesis.

In conclusion, our study established a prognostic ATG signature that can effectively predict DFS for early-stage CRC patients. Meanwhile, our study also revealed the possibility that CRC patients in the high autophagy risk group might suffer tumor relapse *via* anti-immune/anti-inflammatory response. Moreover, higher sensitivity to chemotherapy in the low autophagy risk group was discovered in colorectal cancer-associated cell lines.

## Data Availability

The original contributions presented in the study are included in the article/Supplementary Material, further inquiries can be directed to the corresponding authors.
