# Supplementary figures and images for: Identification of an Autophagy-Related Gene Signature for the Prediction of Prognosis in Early-Stage Colorectal Cancer
Source: Front Genet. 2021 Nov 25;12:755789. doi: 10.3389/fgene.2021.755789 (PMC8657766; doi:10.3389/fgene.2021.755789)

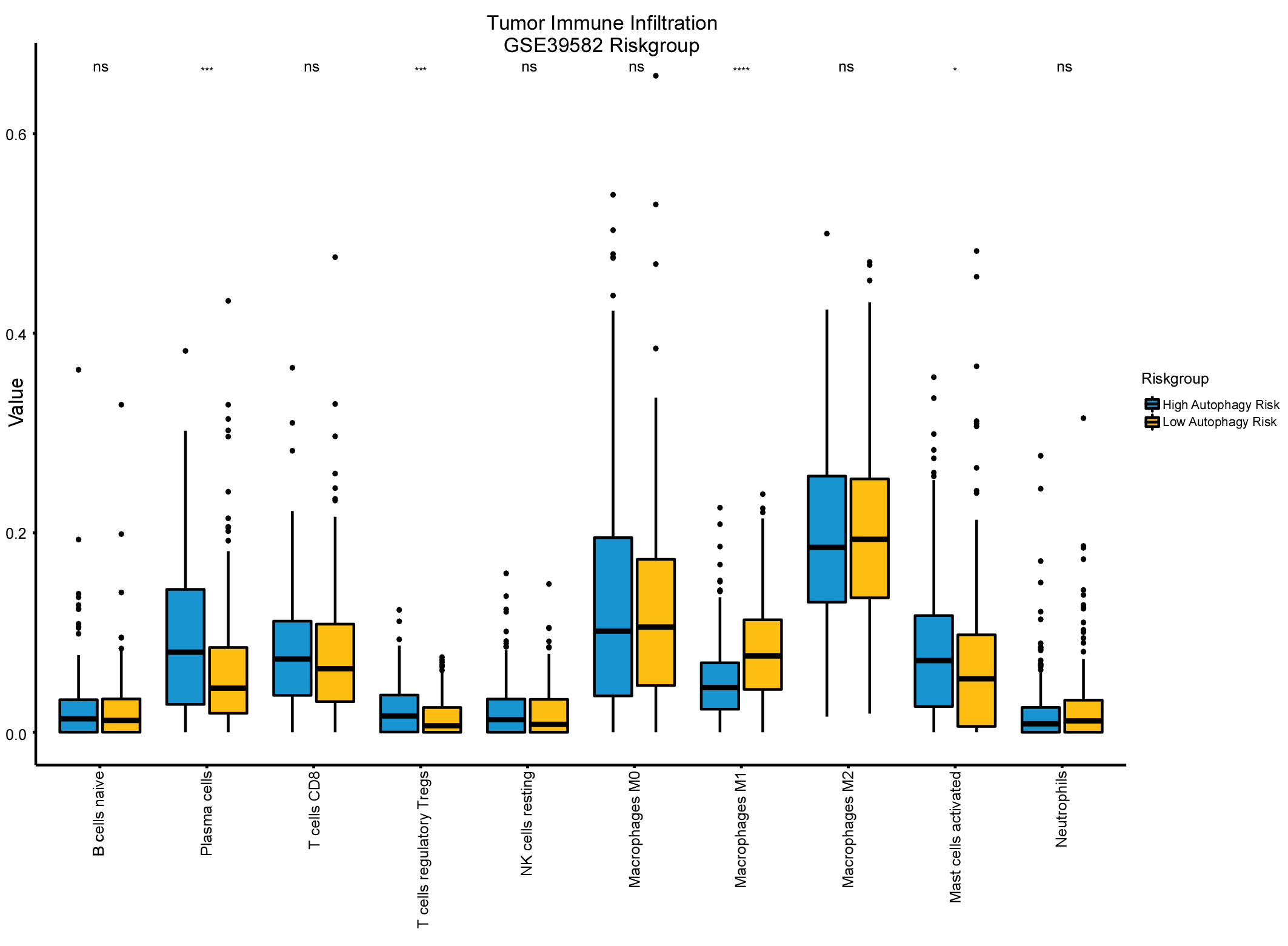

Supplement: Supplementary file 2 [file Image2.TIF]

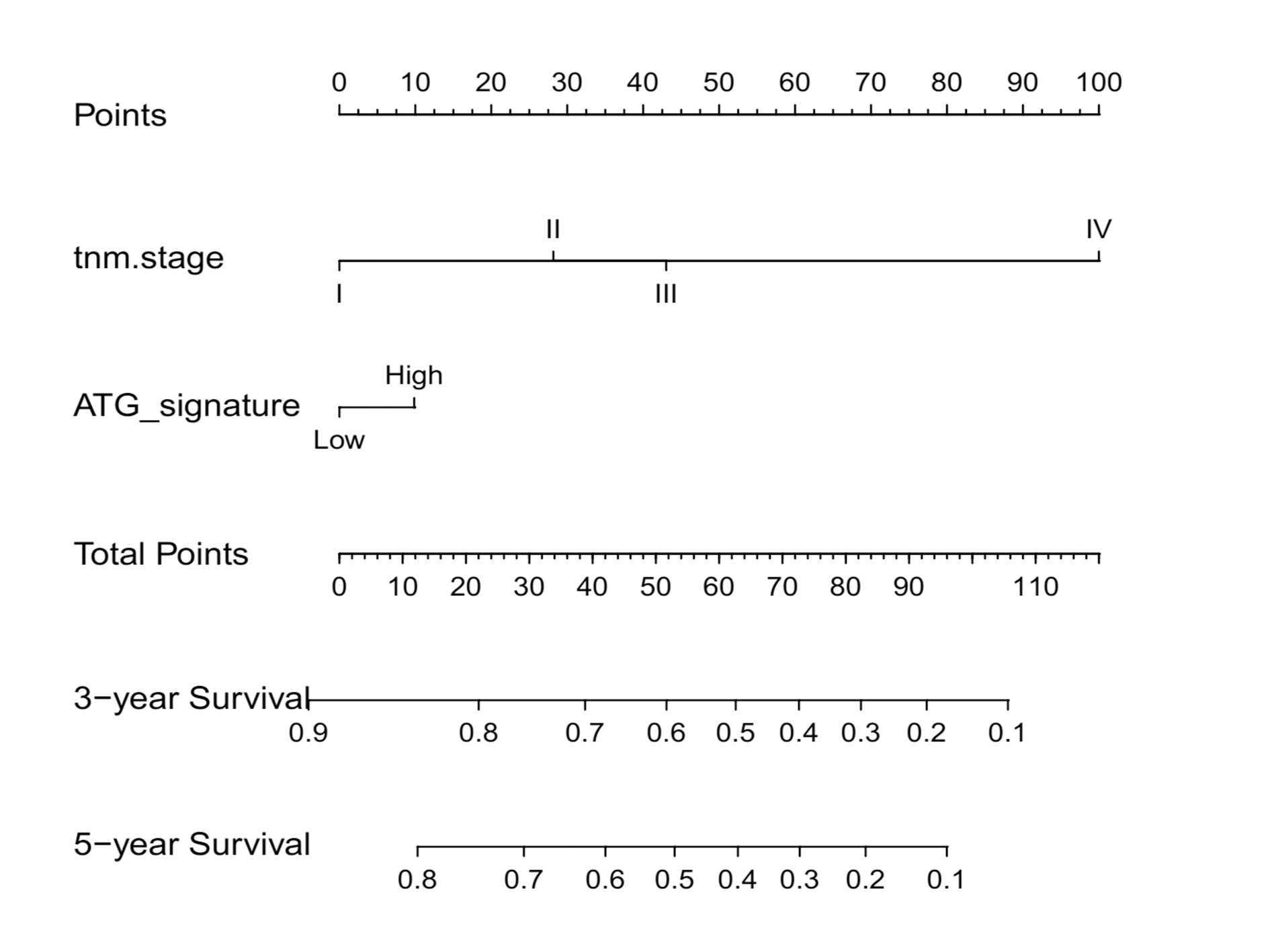

Supplement: Supplementary file 3 [file Image1.TIF]
